# Validation of an ear-worn sensor for gait monitoring using a force-plate instrumented treadmill

**DOI:** 10.1016/j.gaitpost.2011.11.021

**Published:** 2012-04

**Authors:** Louis Atallah, Anatole Wiik, Gareth G. Jones, Benny Lo, Justin P. Cobb, Andrew Amis, Guang-Zhong Yang

**Affiliations:** a The Hamlyn Centre for Robotic Surgery, Institute of Global Health Innovation, Imperial College London SW7 2BZ, UK; b Department of Surgery and Cancer, Imperial College London, UK; c Department of Mechanical Engineering, Imperial College London, UK

**Keywords:** Wearable sensors, Body sensor networks, Accelerometer, Gait cycle, Instrumented treadmill

## Abstract

A force-plate instrumented treadmill (Hp Cosmos Gaitway) was used to validate the use of a miniaturised lightweight ear-worn sensor (7.4 g) for gait monitoring. Thirty-four healthy subjects were asked to progress up to their maximum walking speed on the treadmill (starting at 5 km/h, with 0.5 km increments). The sensor houses a 3D accelerometer which measures medio-lateral (ML), vertical (VT) and anterior–posterior (AP) acceleration. Maximum signal ranges and zero crossings were derived from accelerometer signals per axis, having corrected for head motion and signal noise. The maximal force, measured by the instrumented treadmill correlated best with a combination of VT and AP acceleration (R-squared = 0.36, *p* = 0), and combined VT, ML, and AP acceleration (R-squared = 0.36, *p* = 0). Weight-acceptance peak force and impulse values also correlated well with VT and AP acceleration (Weight acceptance: R-squared = 0.35, *p* = 0, Impulse: 0.26, *p* = 0), and combined VT, ML, and AP acceleration (Weight acceptance: R-squared = 0.35, *p* = 0, Impulse: 0.26, *p* = 0). Zero crossing features on the ML axis provided an accurate prediction of the gait-cycle, with a mean difference of 0.03 s (−0.01, 0.05 confidence intervals).

## 1 Introduction

The ability to analyse gait patterns and identify potentially correctable variables, which may reduce the risk of injury or osteoarthritis, is highly desirable. Force-plate instrumented treadmills may provide such information, but are expensive and limited to laboratory settings. This technical note aims to validate the use of a non-invasive ear-sensor [1] in conjunction with a treadmill to provide a more economic stand-alone method of walking pattern analysis.

Accelerometers offer a lightweight and portable method of gait analysis when compared to conventional techniques such as optoelectronic and force-plate motion. Their use in assessing walking patterns [2], shock absorption during walking and running [3,4], gait cycle [7], and cadence, step length, and symmetry of walking [8], is well documented. Kavanagh et al. [5] demonstrated that tri-axial accelerometers positioned on the head provided smoother oscillations of higher power at low frequencies when assessing walking than those positioned on the trunk. This work aims to validate the use of a head (ear) worn sensor to observe gait cycle, and the relationship between acceleration and force parameters, at different walking speeds.

## 2 Methods

### 2.1 Participants and settings

34 healthy participants (21 men, 13 women) were recruited for this study [age: 28.22 (12.77) years, weight: 76.22 (14.44) kg, height: 1.76 (0.11) m, BMI: 24.58 (3.0)]. Each subject walked on a force-plate instrumented treadmill at speeds of 5, 5.5, 6, 6.5, 7, 7.5, and 8 km/h whilst wearing an e-AR sensor. The device, worn discretely behind the ear, is a small (5.6 cm × 3.5 cm × 1.0 cm) and light-weight (7.4 g) wireless miniaturised sensor containing a tri-axial MEMS accelerometer (ADXL335) which measures acceleration with a range of ±3 g. Analogue to Digital Conversion (ADC) of this data results in the vertical (VT), medio-lateral (ML) and anterior–posterior (AP) direction accelerometer channel outputs ranging from 0 to 4096, representing 0–3 V. The e-AR has previously been used for minute-by-minute energy expenditure prediction [8], and gait monitoring following knee-replacement surgery [9]. A sampling rate of 50 Hz was used throughout this study. The temperature effect is minimised by the housing of the sensor, and an embedded voltage regulator is included to maintain a constant voltage supply.

An Hp Cosmos Gaitway instrumented treadmill consisting of two tandem piezoelectric force-plate units was used during the experiment. The force plates measure vertical ground reaction forces (GRF), and can discriminate between right and left footsteps, facilitating measurement of cadence, impulse peak-forces, weight acceptance rate, stride length and maximum applied force.

### 2.2 Modelling and statistical approach

Walking speed was recorded in real-time during collection of e-AR sensor data. Data for each axis of the accelerometer was fitted with a third-degree polynomial which was then subtracted from the raw data to correct for head motion during walking. Zero-crossings were then calculated per axis, and averaged per speed (per subject). A peak detection algorithm was used to detect the maximum value of the acceleration amplitude signal per stride, and these were subsequently averaged for each walking speed. This averaging ensures that for every person, walking at a certain speed results in two e-AR features per speed: the zero crossing and the maximal amplitude feature.

Multiple linear regression was used to assess the relationship between the e-AR sensor maximum amplitude feature, and the treadmill-derived gait parameters, namely maximal force, weight acceptance rate and impulse (all normalised for subject weight). Bland–Altman plots, 95% confidence intervals of the difference between the two means, and standard error (SE) were used to analyse agreement between gait cycles derived by the e-AR sensor (using zero crossings), and the treadmill. Data processing and statistical analysis were performed using Matlab (Mathworks, Inc., Cambridge, UK).

## 3 Results

Table 1 contains the results of multiple linear regression using the maximal amplitude feature per accelerometer axis (individual axes, two axes, and all three axes) for all speeds and subjects combined. Treadmill features used for comparison were maximal force, weight acceptance rate and impulse (all normalised for subject weight).

*Maximal force*. The best values for R-squared were obtained from a combination of VT/AP axes, and VT/ML/AP axes (these combinations also provided the lowest estimate of error variance).

*Weight-acceptance peak force and impulse*. A combination of VT/AP, and VT/ML/AP axes provided the best R-squared values. Impulse R-squared values were lower than those for weight-acceptance peak force, and maximal force.

**Fig. 1:**
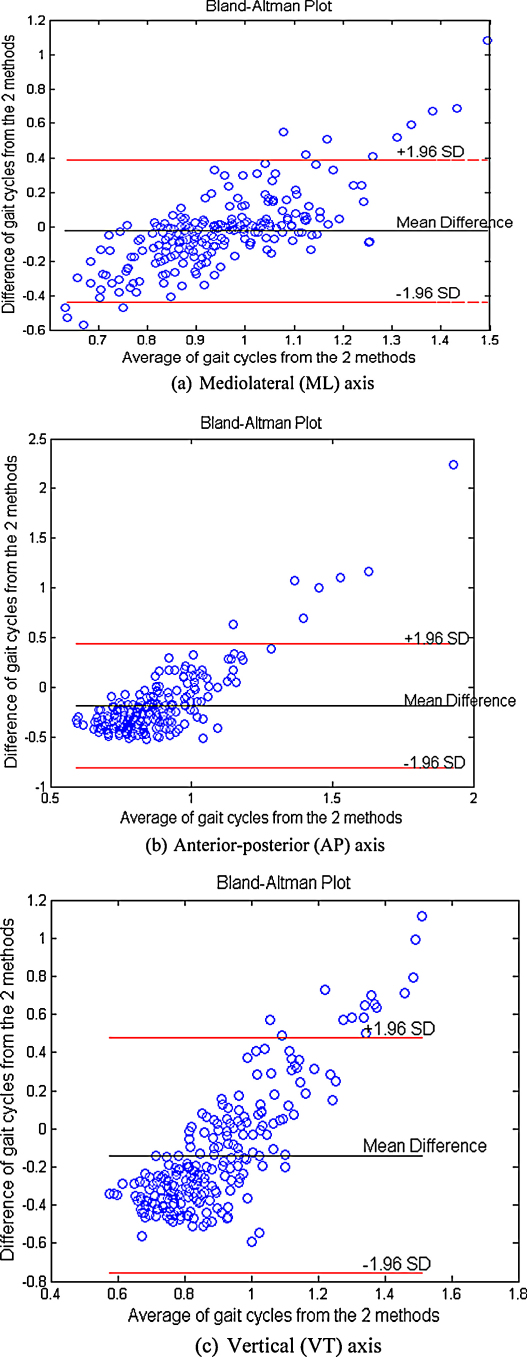
(a–c) Bland–Altman plots for the gait cycle in seconds from both the e-AR sensor and the treadmill. Each point represents a measure of gait cycle per speed for each subject. All subjects are combined in the plot to observe the match between the two methods in calculating gait cycle.

Fig. 1 shows the Bland–Altman plots comparing the gait-cycle parameter predicted from both treadmill and e-AR sensor zero-crossings. The error increases as gait cycles increase, i.e., for lower speeds. At these speed settings, subjects were freer to sway and move their head which probably lead to a few false detections due to zero-crossings detected due to sudden head motion while talking or looking around. However, the values for gait cycle from the ear sensor are very close to the treadmill values (as shown in Table 2), especially for the ML axis where the mean difference is 0.02 s with [−0.01, 0.05] as confidence intervals for the bias.

## 4 Discussion

The survey by Kavannagh et al. [2] summarises the use of accelerometry for gait-feature detection. Related work includes the use of accelerometry to derive shock absorption during walking [3] and running [4] as well as the derivation of tempero-spacial gait parameters such as gait cycle, cadence, step length and symmetry of walking [7]. Although accelerometers have been widely used for gait assessment, this is the first study that validates gait features obtained by a small, fully integrated head-worn accelerometer with instrumented treadmill parameters relating to both force-loading and gait-cycle. These features are indicative of walking patterns and can provide real-time feedback whilst training on a treadmill. Regression models could be developed using this methodology to provide force-loading features for different speeds in real-time. The use of an e-AR sensor in combination with a treadmill is cost effective when compared to the cost of force-plate instrumented treadmills or instrumented gait labs.

Compared to existing sensors that have been used for gait analysis [5,6,10], the e-AR sensor is a lightweight sensor (7.4 g) discreetly positioned behind the ear. It improves compliance, especially during long-term monitoring in a home environment, as well as for continuous assessment of the progress of rehabilitation after knee and hip replacement and activities of daily living.

## Figures and Tables

**Table 1:** Multiple regression analysis the vertical (VT), medio-lateral (ML) and anterior–posterior (AP) acceleration features separately, of each two combined, then all features in the linear regression model. The treadmill parameters considered are maximal force normalised by body weight, weight-acceptance rate (normalised by body weight) and impulse (normalised by body weight).

Treadmill-derived gait parameter	MLR test	R-square Stats	*F*-stat	*P*-value	Estimate of error variance
Maximal force (normalised by body weight)	ML	0.16	43.58	0	0.0168
	AP	0.28	87.36	0	0.0145
	VT	0.21	59.24	0	0.0159
	AP and ML	0.29	47	0	0.0142
	VT and AP	0.36	64	0	0.0128
	VT and ML	0.26	39.86	0	0.0149
	ML, AP and VT	0.36	43	0	0.0129

Weight-acceptance peak force normalised by subject weight	ML	0.15	41.89	0	0.02
	AP	0.28	89.59	0	0.017
	VT	0.19	54.04	0	0.02
	AP and ML	0.29	47.40	0	0.017
	VT and AP	0.35	61.54	0	0.0157
	VT and ML	0.24	36.64	0	0.0183
	ML, AP and VT	0.35	41.09	0	0.0158

Impulse (normalised by weight)	ML	0.06	14.41	0	0.24
	AP	0.08	20.76	0	0.23
	VT	0.24	72.86	0	0.19
	AP and ML	0.09	11.61	0	0.23
	VT and AP	0.26	39.24	0	0.19
	VT and ML	0.24	36.53	0	0.20
	ML, AP and VT	0.26	26.15	0	0.19

**Table 2:** The table shows the number of points analysed per axis (*N*), the mean difference (between the gait cycle from the e-AR sensor and the treadmill), the SD for the difference between the two values, as well as the lower and upper limits of agreement between the two values for gait-cycle, including 95% confidence intervals.

Axis	*N*	Mean difference (95% confidence interval for the bias) in s	Standard deviation (SD) in s	Lower limit of agreement (95% confidence interval for lower limit) in s	Upper limit of agreement (95% confidence interval for upper limit) in s
Medio lateral (ML)	228	0.03 [−0.01, 0.05]	0.21	−0.39 [−0.44, −0.35]	0.45 [0.40, 0.49]
Anterior–posterior (AP)	228	0.19 [0.15, 0.23]	0.32	−0.45 [−0.53, −0.38]	0.83 [0.76, 0.90]
Vertical (VT)	228	0.14 [0.1, 0.18]	0.32	−0.49 [−0.56, −0.42]	0.77 [0.70, 0.84]
