# Supplementary figures and images for: Validation of an ear-worn sensor for gait monitoring using a force-plate instrumented treadmill
Source: Gait Posture. 2012 Apr;35(4):674–6. doi: 10.1016/j.gaitpost.2011.11.021 (PMC3329626; doi:10.1016/j.gaitpost.2011.11.021)

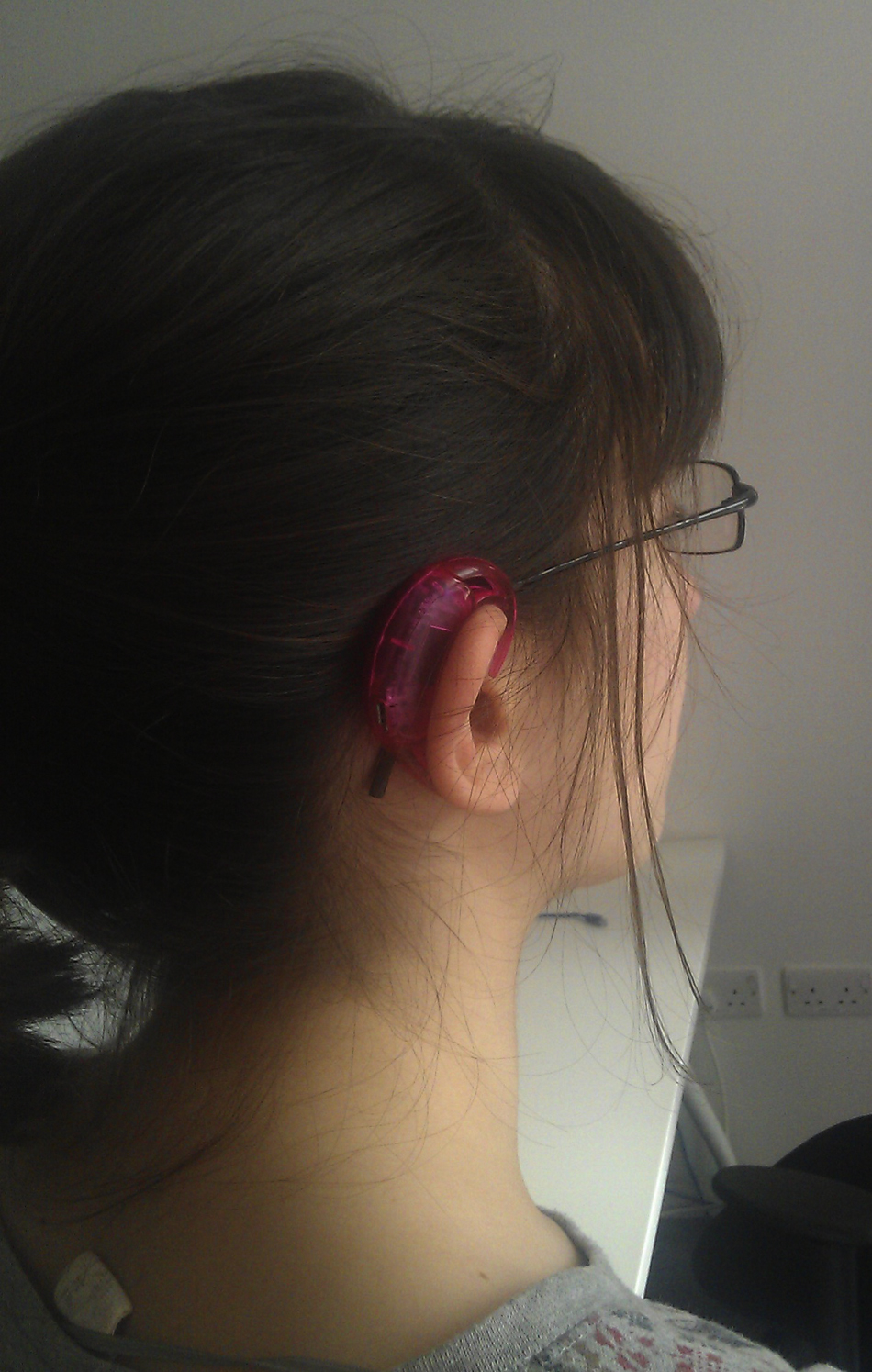

Supplement: Supplementary file 1 [file mmc1.zip › mmc1.jpg]
